# Direct observation of a Fermi liquid-like normal state in an iron-pnictide superconductor

**DOI:** 10.1038/srep12421

**Published:** 2015-07-23

**Authors:** Alona Tytarenko, Yingkai Huang, Anne de Visser, Steve Johnston, Erik van Heumen

**Affiliations:** 1 van der Waals—Zeeman institute, University of Amsterdam Amsterdam XL, 1098 XH, The Netherlands; 2 Department of Physics and Astronomy, University of Tennessee, Knoxville, TN 37996, USA

## Abstract

There are two prerequisites for understanding high-temperature (high-T_*c*_) superconductivity: identifying the pairing interaction and obtaining a correct description of the normal state from which superconductivity emerges. The nature of the normal state of iron-pnictide superconductors, and the role played by correlations arising from partially screened interactions, are still under debate. Here we show that the normal state of carefully annealed electron-doped BaFe_2−*x*_Co_*x*_As_2_ at low temperatures has all the hallmark properties of a local Fermi liquid, with a more incoherent state emerging at elevated temperatures, an identification made possible using bulk-sensitive optical spectroscopy with high frequency and temperature resolution. The frequency dependent scattering rate extracted from the optical conductivity deviates from the expected scaling *M*_2_ (*ω*, *T*) ∝ (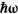)^2^ + (*pπk*_*B*_*T*)^2^ with *p *≈ 1.47 rather than *p* = 2, indicative of the presence of residual elastic resonant scattering. Excellent agreement between the experimental results and theoretical modeling allows us to extract the characteristic Fermi liquid scale *T*_0_* *≈ 1700 K. Our results show that the electron-doped iron-pnictides should be regarded as weakly correlated Fermi liquids with a weak mass enhancement resulting from residual electron-electron scattering from thermally excited quasi-particles.

Strong electronic correlations and Mott physics have played an important role in shaping our understanding of high-*T*_*c*_ superconductivity (HTSC)^1^. With the discovery of the iron-pnictide family of HTSCs a new playground to study correlation effects has emerged^2^. Unlike the cuprate HTSC, the pnictides are properly classified as moderately correlated semi-metals^3^. By studying their normal state properties a new picture has started to emerge^4^ where intra-atomic exchange processes (Hund’s coupling) govern the degree of correlation effects. In the resulting “Hund’s metal” state^5^, Hund’s coupling reduces the propensity towards a strongly correlated Mott insulating state, while simultaneously reducing the coherence temperature below which Fermi liquid (FL) properties emerge. A strong dependence of the nature of this Hund’s metal state on orbital filling has been found, providing a natural explanation for the differences between hole- and electron-doped pnictides^2^,^6^,^7^. Recently, Werner *et al.* showed^8^ that the combined effect of dynamic screening (manifested through a single particle self-energy, Σ(*ω*, *T*)) and orbital occupancy results in a Fermi-liquid like state in electron-doped pnictides, while a spin-freezing transition separates an incoherent metal regime from the FL regime in hole-doped materials (for a more extensive review of the role of Hund’s coupling in the iron-pnictides, see ref. 2). A clear experimental identification of both these regimes is currently lacking. Here, we provide direct experimental confirmation of the Fermi liquid state in the electron-doped case.

Optical spectroscopy is a powerful tool to probe self-energy effects^9^ as a function of frequency and temperature simultaneously. The complex-valued free charge optical conductivity^10^ can be written as


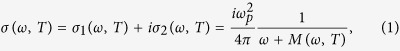


where 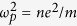 is the plasma frequency and *M* (*ω*, *T*) = *M*_1_ (*ω*, *T*) + *iM*_2_ (*ω*, *T*) is the complex memory function. For a simple Drude metal *M* (*ω*, *T*) = *i*Γ_*D*_ is frequency independent, while interactions beyond simple impurity scattering introduce a frequency and temperature dependence. In the latter case Eq. (1) is referred to as the “Extended Drude model”. The single particle self-energy Σ(*ω*, *T*) thus manifests itself in the free charge carrier response, appearing as a deviation from a classical Drude response. For a local FL with a momentum independent interaction between electrons, Σ(*ω*, *T*) and consequently *M* (*ω*, *T*) (see Methods) follow a universal quadratic dependence on both energy and temperature^11^,^12^,^13^,^14^,


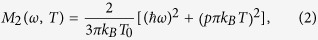


where *k*_*B*_*T*_0_ is an overall energy scale characterising the correlation strength and *p* is a non-universal constant. For a local FL one expects *p* = 2; however, deviations arise in the presence of additional elastic resonant scattering channels^12^. To date the only known example with *p* = 2 is Sr_2_RuO_4_^14^, while *p* ≠ 2 has been reported for several correlated materials^13^,^15^,^16^,^17^,^18^. When applied to the iron-pnictide superconductors, the accurate determination of *M* (*ω*, *T*) is hampered by the presence of low-lying interband transitions. In the following we first show that *M* (*ω*, *T*) extracted for carefully annealed BaFe_2−*x*_Co_*x*_As_2_ single crystals indeed displays the characteristic *ω*, *T*-scaling predicted by Eq. (2). We then introduce an analysis of the complex optical conductivity that represents a direct confirmation of the Fermi liquid normal state of these electron doped iron-pnictides. This is made possible by the 2 K temperature resolution in our experiments, which allows us to compare the detailed frequency *and* temperature dependence with similar resolution.

## Results

**Figure 1:**
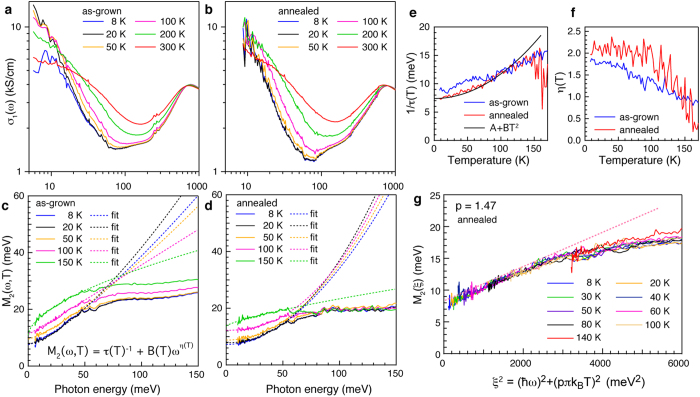
Experimental determination of Fermi liquid behaviour in electron doped BaFe_2_As_2_. (**a**,**b**) Comparison of the real part of the optical conductivity σ_1_ (*ω*) for as-grown (T_*c*_* *≈ 18 K) and annealed (T_*c*_* *≈ 25 K) Ba_1.8_Fe_0.2_Co_2_As_2_ at selected temperatures. The most significant annealing induced change is a reduction of a broad incoherent background that is most clearly seen by the deeper minimum around 70 meV separating the free charge and inter-band optical conductivity. (**c**,**d**) Imaginary part of the memory function, *M*_2_ (*ω*, *T*), revealing the difference in free charge response for as-grown and annealed crystals. The memory function is obtained by subtracting the full interband response as discussed in the text and SOM. Dashed curves indicate fits made using the fitting function indicated in panel **c**. (**e**) Temperature dependence of the static scattering rate 1/*τ* (*T*) obtained from the fits in panels (**c**,**d**). For the annealed crystal 1/*τ* (*T*) displays a T^2^ behaviour below *T*~100 K as indicated by the fit. (**f**), Temperature dependence of the exponent, *η* (*T*), extracted from the fits in panel (**c**,**d**). The exponent for the annealed crystal shows *ω*^2^ dependence in the same temperature range where 1/*τ* (*T*) has a *T*^2^ temperature dependence. At higher temperatures a clear deviation from Fermi liquid behaviour is found. (**g**) Scaling collapse obtained by plotting *M*_2_ (*ω*, *T*) as *M*_2_ (*ξ*), where *ξ*^2^ = (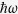)^2^ + (1.47*πk*_*B*_*T*)^2^. Above *ξ*^2^* *≈ 2500 meV^2^ the scaling deviates from the universal Fermi liquid behaviour which is indicated by the dashed pink line.

The in-plane optical conductivity of as-grown and annealed BaFe_1.8_Co_0.2_As_2_ (see Methods) are shown in Fig. 1a,b, respectively. After annealing we observe a decrease in the depth of the minimum around 70 meV separating the free-charge and interband optical conductivity. This arises from a reduction of a broad incoherent response associated with high energy interband processes, rather than from changes in the free-charge response (see Supplementary Online Material, SOM). A spectral weight analysis (see SOM) for both crystals gives *ω*_*p*_* *≈ 1.4 eV and a contribution of interband transitions to the low energy dielectric constant *ε*_∞,*IR*_* *≈ 100. These similarities indicate that annealing does not significantly change the overall electronic structure (such as a chemical potential shift) or high-energy optical properties.

### Experimental signatures of the Fermi liquid state

Subtle changes in the free charge carrier response are more easily analysed in terms of equations (1) and (2), but the extended Drude model analysis assumes that interband transitions do not contribute to the optical conductivity in the energy range of interest. The multi-band nature of the pnictides complicates the extraction of *M* (*ω*, *T*) since inter-band processes have a significant contribution to the optical conductivity^19^,^20^,^21^,^22^. In the SOM we describe the procedure used to extract the memory functions and its range of validity. We find that even though the determination of the memory functions comes with uncertainty at higher energies, at low energies (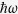 ≤ 50 meV) interband transitions only weakly affect the frequency dependence. In the following we subtract the full frequency dependence of the interband response as outlined in the SOM; however, we note that our conclusions remain the same when alternative methods for accounting for the interband transitions are applied.

The frequency and temperature dependence of the imaginary part of the memory function *M*_2_ (*ω*, *T*), shown in Fig. 1c,d for the as-grown and annealed crystal, respectively, indicates the presence of residual interactions beyond a classical Drude response. We fit both datasets with a power-law form *M*_2_ (*ω*, *T*) = 1/*τ* (0, *T*) + *B* (*T*) *ω*^*η*(*T*)^, where 1/*τ* (0, *T*) is the zero-frequency scattering rate and *η* (*T*) = 2 is expected for a FL. These parameters are determined independently at each temperature. The temperature dependence of 1/*τ* (0, *T*) and *η* (*T*) are displayed in Fig. 1e,f for both the as-grown and annealed crystal. We find that the annealed crystal displays characteristic FL behaviour with 1/*τ* (*T*) ∼ *T*^2^ (Fig. 1e) and *η* (*T*) ~ 2 (Fig. 1f) over a large range of energy (10 meV ≤ 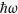 ≤ 50 meV) and temperature (8 K ≤ *T* ≤ 100 K). We further find that the prefactor *B* (*T*) is temperature independent in the same temperature range as is expected from Eq. (2) (see SOM). The as-grown crystal on the other hand does not display FL behaviour. Instead, the zero frequency scattering rate follows a more linear temperature dependence, while the frequency exponent *η* (*T*) < 2. Given the approximate *T*^2^ and *ω*^2^ dependence of the memory function apparent in Fig. 1e,f, we test whether the scaling form of Eq. (2) applies to the annealed crystal. Figure 1g demonstrates that *M*_2_ (*ω*, *T*) indeed follows a universal FL scaling as function of the scaling variable *ξ*^2^ = (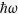)^2^ + (*pπk*_*B*_*T*)^2^, with *p *≈ 1.47 (see SOM).

We highlight three deviations from universal FL behaviour that can be discerned in Fig. 1g. First, universal FL behaviour disappears above 100 K. Second, for *ξ*^2^ ≥ 2500 meV^2^, *M*_2_ (*ξ*) changes slope, as indicated by the dashed pink line, signalling a crossover to a nearly energy independent *M*_2_ (*ω*, *T*) for 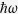 ≥ 50 meV (Fig. 1d). Third, *p* = 1.47 rather than 2, indicating that an additional elastic contribution is present beyond residual electron-electron scattering. We note that the precise value of *p* determined by collapsing the data on a universal curve comes with some uncertainty as it depends on the assumed strength and frequency dependence of the interband contribution (see SOM).

### Fermi liquid signatures in the optical conductivity

Our analysis provides compelling evidence that the normal state of BaFe_1.8_Co_0.2_As_2_ below 100 K is properly classified as a FL. We emphasise that the specific method of accounting for interband processes does not alter the conclusion that the low frequency and temperature dependence of *M*_2_ (*ω*, *T*) follows *ω*^2^ and *T*^2^ scaling (see SOM). In contrast, the same analysis applied to the as-grown crystal does not show such clear signatures of FL behaviour, despite its similar plasma frequency and high-energy optical properties. Nevertheless, the determination of the parameters characterising the Fermi liquid state using the extended Drude analysis remains sensitive to the choice for the interband contribution. To fortify our conclusions, and to determine the characteristic properties of the Fermi liquid state more accurately, we now turn our attention to an analysis of the complex optical conductivity, which provides a more direct comparison between theory and experiment and does not require a model specific choice for the interband processes.

Berthod *et al.* showed^11^ that in a local FL a dome is defined by the locus of points where *σ*_1_ (*ω*, *T*) = *σ*_2_ (*ω*, *T*), which bounds a ‘thermal’ regime in which FL behaviour emerges. Zero crossings signalling the presence of a dome have been clearly observed^14^ in Sr_2_RuO_4_ at low temperatures. Despite the clean Fermi liquid behaviour, exemplified in that case by *p* = 2, these authors found that at elevated temperatures deviations from the predicted dome shape appeared, which they linked to the increasing importance with increasing temperature of ‘resilient’ quasi-particles. This observation provides the means to make a direct comparison between the optical conductivity and theoretical calculations, where one does not have to resort to making the decompositions involved in the extended Drude analysis presented in Fig. 1. To facilitate a direct comparison between experiment and theory we introduce the function Δ*σ* (*ω*, *T*) ≡ *σ*_1_ (*ω*,*T*) − *σ*_2_ (*ω*, *T*), which is readily obtained from experimental data and also from calculations of the optical conductivity. For the particular case of a local FL, the function Δ*σ* (*ω*, *T*) has the property that it is negative in the thermal regime where characteristic FL behaviour should be observed, while it is positive in the incoherent and Drude-like regimes. Moreover, the zeros of this function correspond to the “dome” derived by Berthod *et al.*. Δ*σ* (*ω*, *T*) thus allows us to examine the full, complex optical conductivity and search for zero-crossings where *σ*_1_ (*ω*, *T*) = *σ*_2_ (*ω*, *T*).

**Figure 2:**
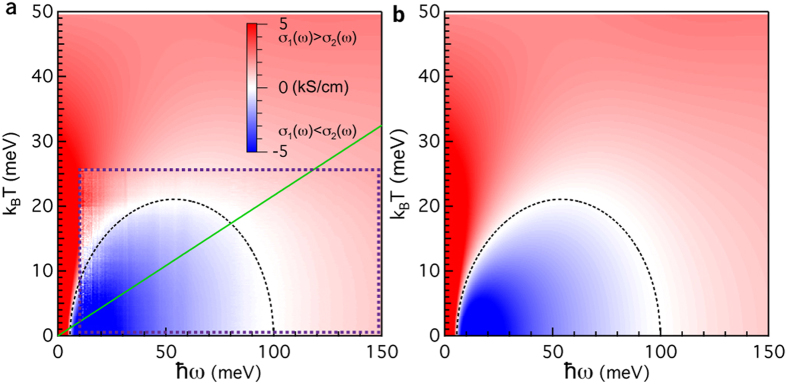
Fermi liquid behaviour of the optical conductivity: theory vs. experiment. (**a**) (*ω*, T) dependence of Δ*σ* (*ω*, *T*) ≡ *σ*_1_ (*ω*, *T*)−*σ*_2_(*ω*, *T*). The experimental data is bounded by the purple dashed box, while the background image is the same as in panel **b**. Colour is used to indicate the magnitude of Δ*σ* (*ω*, *T*), with red indicating the dissipative regime (*σ*_1_ (*ω*, *T*) > *σ*_2_ (*ω*, *T*)) and blue indicating the inductive regime (*σ*_1_ (*ω*, *T*) < *σ*_2_ (*ω*, *T*)). The colour scale is chosen such that the boundary between these two regimes, where (*σ*_1_ (*ω*, *T*)* *≈ *σ*_2_ (*ω*, *T*), or Δ*σ* (*ω*, *T*)* *≈ 0, appears as white. This dome of zeroes can be reproduced using the approximate expression *T*_∞_ (*ω*). The green line indicates the crossover temperature 1.47*πk*_*B*_*T* = 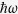, below which Fermi liquid behaviour can be expected. (**b**), Same as in panel **a**, but calculated from the Allen-Kubo formula for the optical conductivity using a Fermi liquid self-energy with parameters derived from the experimental data of Fig. 1. The dashed semi-circle is the same as in panel **a**.

**Figure 3:**
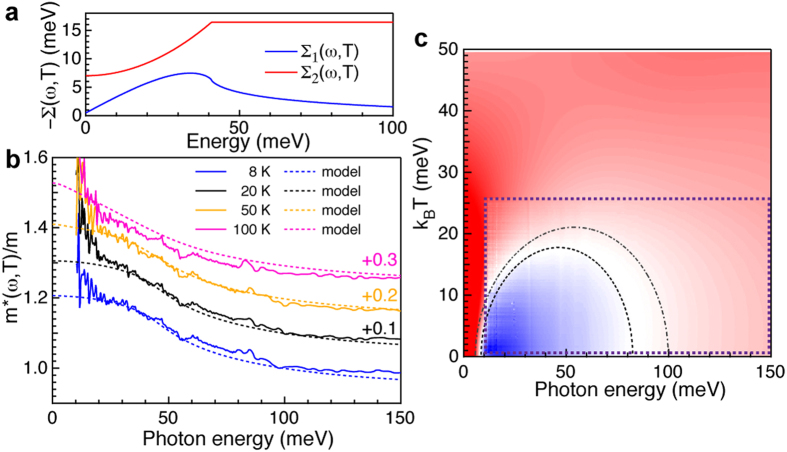
Self-energy, mass enhancement and Fermi liquid properties of as-grown BaFe_1.8_Co_0.2_As_2_. (**a**) single particle self-energy, Σ(*ω*, 8 K), extracted from the optical data. The cutoff energy in Σ_2_ (*ω*, *T*) at *ω*_*c*_ = 41 meV introduces a finite slope of Σ_1_(*ω*, *T*) at lower energy and a corresponding mass enhancement. (**b**), energy and temperature dependence of the effective mass of the annealed crystal corresponding to the optical scattering rate of Fig. 1d. The experimental mass enhancement is shown with solid lines, while the mass enhancement calculated from the Allen-Kubo formula is shown as dashed lines. The effect of the cutoff energy *ω*_*c*_ results in a mass enhancement m^*^/*m* (*ω* → 0)* *≈ 1.2. Note that the data and fits are offset from their actual value with increments of 0.1 at successive temperatures above the 8 K curves. (**c**) (*ω*, *T*) dependence of Δ*σ* (*ω*, *T*) for the as-grown crystal. The dome of zero-crossings is smaller compared to the annealed crystal. This difference is highlighted by the dashed (as-grown) and dashed-dotted (annealed) semi-circles calculated from *T*_∞_ (*ω*). The dashed semi circle is calculated using the same parameters as in Fig. 2 except for Γ_0_* *≈ 8 meV and *p *≈ 1.34.

In Fig. 2a, Δ*σ* (*ω*, *T*) is displayed as a false color plot for the annealed crystal. In Fig. 2a, blue represents Δ*σ* (*ω*, *T*) < 0, while red indicates Δ*σ* (*ω*, *T*) > 0. Δ*σ* (*ω*, *T*) = 0 is indicated in white. The most striking feature of Fig. 2a is a clear dome of zero crossings, closely resembling the dome predicted by Berthod *et al.*. In Fig. 2b we display calculations of Δ*σ* (*ω*, *T*), assuming a FL self-energy (see Methods and SOM for calculation details). Motivated by the saturation of *M*_2_ (*ω*, *T*) above 50 meV (Fig. 1d), we have introduced a cutoff *ω*_*c*_ above which the imaginary part of the single particle self-energy, Σ_2_ (*ω*, *T*), is constant (see Fig. 3a and Methods) and a high energy cutoff *D*. The calculated Δ*σ* (*ω*, *T*) is in excellent agreement with the experimental data. As input for the calculation we have used several experimentally available parameters, namely *ω*_*p*_* *≈ 1.4 eV, Γ_0_ = *M*_2_ (*ξ* → 0)* *≈ 7 meV and *p *≈ 1.47. The cutoffs *ω*_*c*_* *≈ 41 meV and *D *≈ 1 eV are motivated below. In addition to the free charge response, we also include the frequency dependent interband response from Supplementary Table S1. The only remaining free parameter, *T*_0_* *≈ 1700 K, is determined by two criteria: (i) the maximum of the dome of zero crossings (at 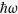
* *≈ 55 meV) and (ii) the low temperature zero-crossing at 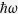
* *≈ 100 meV. To facilitate the estimation of *T*_0_, we derive an approximate analytical expression, *T*_∞_ (*ω*) (see Methods and SOM), for these zero-crossings taking an energy independent interband response (e.g. *ε*_∞_) into account. The consistency between *T*_∞_ (*ω*), the data, and the calculation (which includes the full frequency dependence of the interband response) shows that the details of the interband response are unimportant for obtaining this level of agreement.

### Characteristic Fermi liquid properties of Co-doped BaFe_2_As_2_

The deviation from scaling in Fig. 1e–g around 100 K signals a crossover temperature where 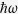 ≤ *pπk*_*B*_*T*, above which an incoherent regime emerges^12^,^11^. This suggests a natural cutoff 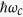
* *≈ 1.47*πk*_*B*_*T* with *T *≈ 100 K, resulting in *ω*_*c*_* *≈ 41 meV. The cutoff *D *≈ 1 eV is less critical but is motivated by the value of *T*_0_. Dynamical Mean Field Theory (DMFT) calculations for a single band Hubbard model^11^ indicate that *k*_*B*_*T*_0_* *≈ 0.57*δW* where *W* is half the bandwidth and *δ* is the carrier density. This yields *W *≈ 1.3 eV in our case, which is reasonable compared to combined density functional theory and DMFT (e.g LDA + DMFT) estimates^8^.

Apart from the saturation in Σ_2_ (*ω*, *T*), *ω*_*c*_ also introduces a frequency dependence in Σ_1_ (*ω*, *T*) (see Fig. 3a), which should be manifest as a frequency dependent mass enhancement m^*^/*m* (*ω*, *T*)≡1 + *M*_1_(*ω*, *T*)/*ω* in the free charge response. Figure 3b shows excellent agreement between m^*^/*m*(*ω*, *T*) extracted from experiment and the theoretical calculation where m^*^/*m* (*ω* → 0, *T*)* *≈ 1.2. This value is consistent with a modest m^*^/*m *≈ 1.8 predicted by LDA + DMFT calculations for this level of electron doping^8^. The experimental data leaves some room for additional mass enhancement resulting from boson exchange processes (such as phonons or spin-fluctuations) below 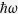
* *≈ 10 meV, although it is difficult to make a quantitative statement on their strength due to the low signal-to-noise at low energy. More importantly, the energy dependence of the mass-enhancement introduced through the cutoff in our self-energy rules out the presence of a significant boson exchange spectrum for 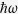 ≥ 10 meV. We emphasise that the calculated mass enhancement is based on an analysis of the optical conductivity, while the experimental mass enhancement is determined using the extended Drude analysis presented in Fig. 1. The excellent agreement between the experimental and calculated m^*^/*m*(*ω*, *T*) therefore serves as a confirmation of the analysis presented in Fig. 1.

## Discussion

To conclude we discuss the deviation of *p* from the FL value *p* = 2. The most likely origin appears to be scattering of quasi-particles on weak, localised magnetic moments^12^. Such localised moments could be associated with the presence of Co impurities in the Fe lattice, although no local moment has been detected for Co impurities in BaFe_2_As_2_^23^. Regardless the origin, this resonant elastic term has a strong influence on the normal state properties. Figure 3c displays Δ*σ* (*ω*, *T*) for the as-grown crystal, displaying a suppressed dome of zero-crossings compared to the annealed crystal. The dashed semi-circle is calculated using exactly the same parameters as for the annealed case, except for a slightly higher Γ_0_* *≈ 8 and *p* = 1.34. This smaller value of *p* corresponds to a two-fold stronger elastic term in the single particle self-energy Σ (*ω*, *T*), indicating that annealing strongly reduces the influence of this scattering channel. Given the concomitant change in superconducting critical temperature, we suggest that this scattering channel could be pair-breaking, possibly providing an interesting direction for future work.

## Methods

### Method subsection

A large 4 × 5 × 0.1 mm^3^ single crystal of BaFe_1.8_Co_0.2_As_2_, grown from self-flux, was cut into two pieces and one piece was subsequently annealed for 75 hours at 800 °C. The dc resistivity and dc susceptibility show an increase of the critical temperature Δ*T*_*c*_/*T*_*c*_* *≈ 0.3 upon annealing, while the overall value of the resistivity decreases. Further details of the experiments are presented in the SOM. The theoretical formalism is based on the Allen-Kubo formula for the optical conductivity^24^,


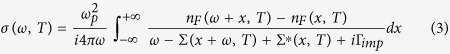


which we evaluated numerically. The imaginary part of the single particle self-energy appearing in the denominator is given by^12^,


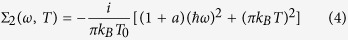


for a local Fermi liquid with an additional elastic resonant scattering contribution (note *a* = (*p*^2^ − 4)/(1 − *p*^2^)^12^). Such an energy and temperature dependent Σ_2_ (*ω*, *T*) results at low temperature in an imaginary memory function^12^,^11^,


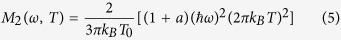


Together with equation (1) for the optical conductivity and an interband contribution characterised by an energy independent value *ε*_∞,*IR*_, equation (5) leads to the following expression for the dashed semi-circle displayed in Fig. 2:
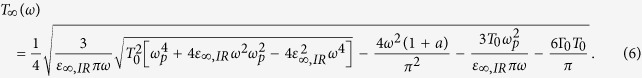
For a derivation and further details see the SOM. The parameters used to calculate the dashed semi-circles in Fig. 2a,b are the same as for the full calculation except for *ε*_∞,*IR*_* *≈ 100. For the full calculation of equation (3), we introduce two cutoff’s, *ω*_*c*_ and *D* in equation (4) such that the imaginary part of the self-energy is given by Σ_2_ (*ω*) ∝ *ω*^2^ for |*ω*| < *ω*_*c*_; 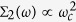 for |*ω*| ∈ [*ω*_*c*_, *D*]; and Σ_2_ (*ω*) = 0 otherwise. In the SOM we derive analytical expressions for the real part of the self-energy obtained from Kramers-Kronig transformation.

## Additional Information

**How to cite this article**: Tytarenko, A. *et al.* Direct observation of a Fermi liquid-like normal state in an iron-pnictide superconductor. *Sci. Rep.* **5**, 12421; doi: 10.1038/srep12421 (2015).

## Supplementary Material

Supplementary Information
