# Supplementary material for: Direct observation of a Fermi liquid-like normal state in an iron-pnictide superconductor
Source: Sci Rep. 2015 Jul 23;5:12421. doi: 10.1038/srep12421 (PMC5378886; doi:10.1038/srep12421)
Supplement: Supplementary Information [file srep12421-s1.pdf]

Supplementary material: Direct observation of a Fermi liquid-like normal state in an iron-pnictide superconductor.

Alona Tytarenko,[1] Yingkai Huang,[1] Anne de Visser,[1] Steve Johnston,[2] and Erik van Heumen[1,*]

[1]*van der Waals - Zeeman institute, University of Amsterdam, 1018 XL Amsterdam, the Netherlands*
[2]*Department of Physics and Astronomy, University of Tennessee, Knoxville, U.S.A.*

(Dated: April 23, 2015)

I. TRANSPORT EXPERIMENTS

The crystal used in this study was grown from a self-flux method. Its chemical composition has been determined with electron probe microanalysis, resulting in the determination of a Co concentration, $x = 0.195$. We subsequently cut the crystal into two pieces and annealed one piece for 75 hours at 800 °C. The dc resistivity and susceptibility were measured for both pieces, see Fig. S1. The resistivity shows a significant enhancement of the critical temperature

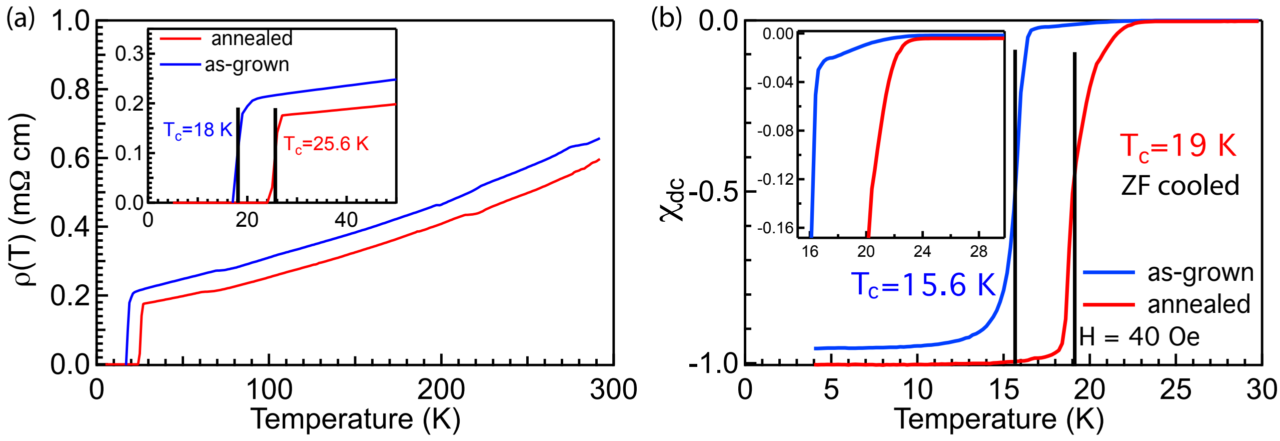

FIG. S1: (Color online) **Transport and magnetisation measurements.** Two crystals were measured: as-grown (blue) and annealed (red) crystals. (a) dc resistivity with an approximate T^2 dependence up to 300 K. The little kinks at 70 K and 220 K are experimental artefacts. The inset shows an enlarged view of the superconducting transitions to the zero-resistance state. (b) dc susceptibility measured in a 40 Oersted field. As the inset shows the onset of the transition takes place at significantly higher temperature compared to the temperature where the full volume of the crystal becomes superconducting.

from 18 K for the as-grown crystal to 25.6 K in the annealed crystal. The dc susceptibility shows an onset to superconductivity at similar temperatures, but the full Meisner volume is obtained at somewhat lower temperatures.

II. REFLECTIVITY DATA

The in-plane reflectivity for both crystals was obtained in the range between 5 meV and 4.6 eV using a Bruker vertex 80v Fourier-Transform infrared spectrometer. In all experiments unpolarised light was used and experiments were performed at near-normal incidence (8 degrees) to the ab-plane. Crystals were mounted on a tapered copper cone, preventing unwanted reflections from copper, and were cleaved before inserting them into a home-built UHV cryostat. The cryostat has a rigid sample support decoupled from the cryostat cold finger with a copper braid. This enables optical experiments as function of temperature in which the sample position does not change as temperature is changed. To test sample position stability of the cryostat a HeNe laser was reflected from a mirror mounted on the sample position and the movement of the reflected beam over a distance of 5 meters was measured, confirming

*Electronic address: e.vanheumen@uva.nl

that the sample orientation changed less than 0.01 ° between room temperature and the base temperature of 8 K. In the experiments an aperture size was chosen such that the light spot slightly overfills the sample surface. All experiments were performed under UHV conditions with a pressure at room temperature of order $5 \cdot 10^{-9}$ mbar. A wedged, CVD grown diamond window was used in all experiments. To cover the full energy range, experiments were repeated several times using a series of detectors and beamsplitters. In order to obtain an accurate absolute value of the reflectivity we evaporated metallic films on our samples -itin-situ. Au was used in the far to mid-infrared range (3 meV - 0.75 eV). Silver was used in the mid-infrared to visible range (0.4 - 2.85 eV) and Al in the near-infrared to ultra-violet range (0.75 - 4.6 eV). In the near-infrared to visible range measurements were performed in 3 steps. In the first step the reflection of the sample surface was measured. In the second step Ag was evaporated *in-situ* on the sample surface without making any adjustments to the set-up. Finally in the third step Al was evaporated *in-situ* on the sample surface. For each of these steps the full temperature dependence was measured by collecting 1 spectrum per minute while cooling down with a constant rate of 1.5 K per minute, followed by a similar warming measurement. By combining both warming and cooling measurements we thus obtained 1 spectrum every 2 K. By comparing ratios of Ag and Al spectra to published literature results we obtained standardized reference spectra as function of temperature used to determine the reflectivity in the entire measured frequency range. Silver turned out to be particularly useful in the photon range around 1.2 eV where Al has a weakly temperature dependent interband transition. The resulting reflectivity is shown in figure S2 for selected temperatures. For the as-grown crystal we

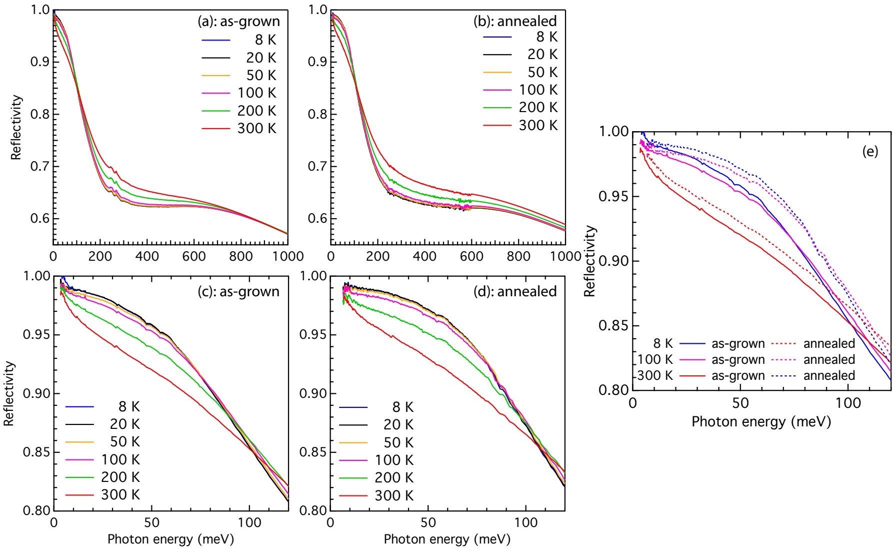

FIG. S2: (Color online) **Reflectivity experiments.** Reflectivity of the as-grown (a,c) and annealed (b,d) crystals at selected temperatures.

observe the opening of the superconducting gap below 18 K as can be seen in panel S2c. The somewhat smaller crystal size (2 x 1 x 0.1 mm^3) for the annealed crystal complicated the accurate determination of the reflectivity below 10 meV most likely due to diffraction effects becoming important at these longer wavelengths. Figure S2e compares the low frequency reflectivity of the annealed and as-grown crystals. Based on the higher reflectivity of the annealed crystal one can immediately observe that the overall scattering rate has decreased, assuming that the charge carrier concentration has not significantly changed. In a first step the reflectivity data is modelled using a Drude-Lorentz model with parameters optimized by a least-square Levenberg-Marquardt routine [1]. We tried different models to determine the robustness of the modelling, as we want to use it later on in the extended Drude analysis. The Drude-Lorentz models presented in table S1 for both crystals, (i) give the lowest χ^2, (ii) consistently describe our optical data at all temperatures and (iii) are nearly identical for both crystals as one might expect. Based on this model and the full reflectivity data we use a variational dielectric function routine developed in [2] to extract the optical conductivity, which is shown at selected temperatures in Fig. 1a,b of the article.

III. DRUDE-LORENTZ MODEL AND INTERBAND TRANSITIONS

In order to make the spectroscopic fingerprints of the effects of annealing more quantitative we first turn to a standard Drude-Lorentz modelling of the data. The decomposition of the optical conductivity of both as-grown and

TABLE S1: **Parameters of the Drude-Lorentz oscillators.** The high-frequency dielectric constant $\epsilon_\infty \approx 9$. ω_j is the centre frequency of an oscillator, ω_{pj} its area (Drude terms) or oscillator strength and γ_j is the width. All values reported correspond to T 40 K data.

as-grown								
j	1	2	3	4	5	6	7	8
$\hbar\omega_j$ (eV)	0	0	0.01	0.12	0.67	0.92	1.6	1.82
$\hbar\omega_{pj}$ (eV)	1	0.96	0.42	0.59	2.49	1.96	14.56	3.65
$\hbar\gamma_j$ (eV)	0.005	0.065	0.01	0.12	0.58	0.73	14.55	1.84
annealed								
$\hbar\omega_j$ (eV)	0	0	0.011	0.13	0.68	0.87	1.6	
$\hbar\omega_{pj}$ (eV)	1.1	0.8	0.51	0.72	1.28	1.62	14.95	
$\hbar\gamma_j$ (eV)	0.0034	0.064	0.01	0.13	0.39	0.72	9.48	

annealed crystals in Drude and Lorentz terms is given in table S1. We find that the intraband contribution can be described by two relatively narrow Drude terms and a low energy Lorentz oscillator. The incoherent background extending to low energy is captured by a high energy oscillator (labeled as nr. 7 in table S1). Upon annealing the width of this oscillator decreases, resulting in a much weaker contribution at low energy. This effect can be clearly seen in the optical conductivity data by comparing the depth of the minimum (at 70 meV) separating the intra- and interband response. Apart from this difference in the incoherent background the Drude-Lorentz models are nearly identical, indicating that the annealing results in a rather subtle change in the intraband response. The optical conductivity of the annealed crystal at 40 K and its decomposition in terms of the oscillators from Table S1 is shown in Fig. S3.

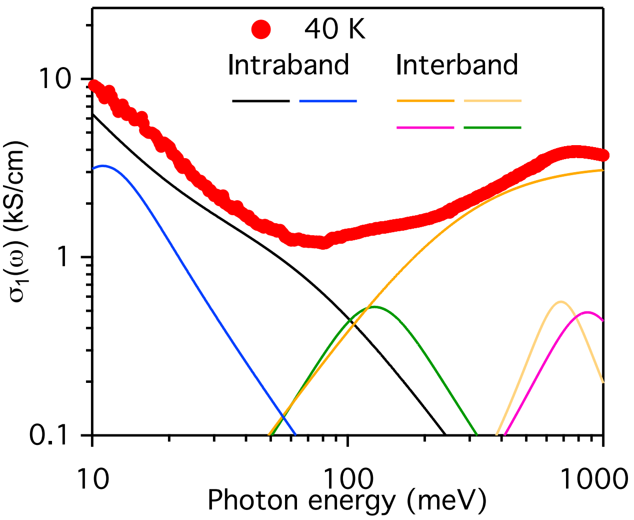

FIG. S3: (Color online) **Decomposition of the optical conductivity.** Experimental spectrum for the annealed crystal at 40 K (symbols) and the decomposition in oscillators (solid lines) corresponding to the parameters given in table S1. Note that the two Drude components are plotted as a single curve (black).

IV. SPECTRAL WEIGHT ANALYSIS

In order to determine the plasma frequency for both crystals we plot in figure S4 the spectral weight as determined from the experimental optical conductivity. The spectral weight is obtained by integrating the real part of the optical conductivityover frequency up to a cutoff frequency ω_c:

$$SW(\omega_c, T) = \int_0^{\omega_c} \sigma_1(\omega, T) d\omega \qquad \text{(S1)}$$

For both crystals we find that the integrated spectral weight is nearly temperature independent for $\omega_c \approx 100$ meV, corresponding roughly to the minimum in the optical conductivity presented in Fig. 1a,b of the main text. At this

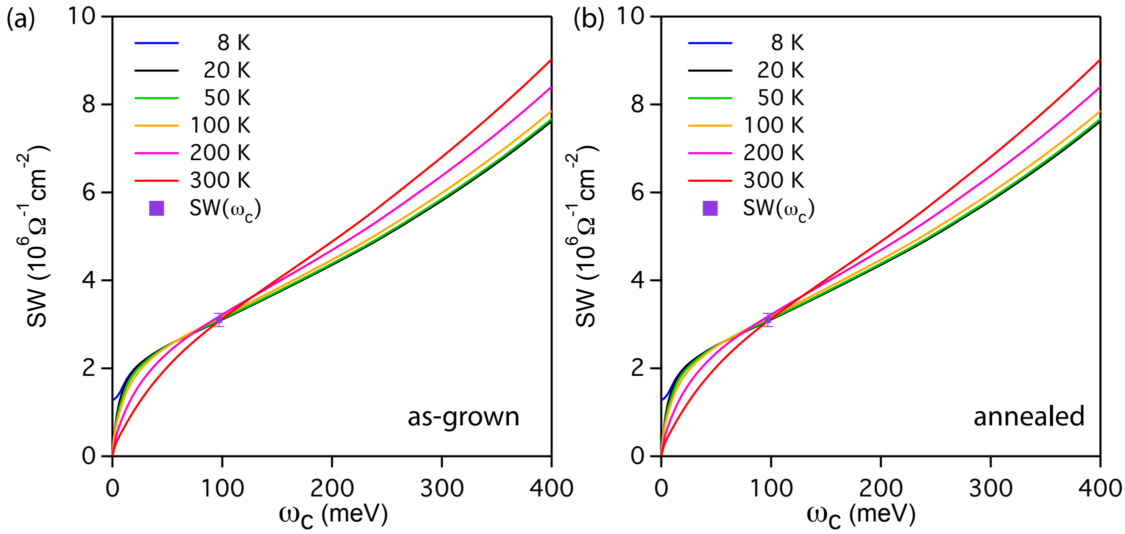

FIG. S4: (Color online) **Spectral weight analysis.** Spectral weight of the as-grown (a) and annealed (b) crystals at selected temperatures.

point the integrated spectral weight $SW(\omega_c, T) \approx 3.1{\pm}0.15 \cdot 10^6\ \Omega^{-1}cm^{-2}$ (as-grown) and $SW(\omega_c, T) \approx 3.4{\pm}0.15 \cdot 10^6\ \Omega^{-1}cm^{-2}$ (annealed). If we assign this spectral weight in both cases entirely to the intraband response we can calculate the plasma frequencies to be $\omega_p \approx 1.35$ eV (as-grown) and $\omega_p \approx 1.4$ eV (annealed). Given the error bar on the estimation of $SW(\omega_c, T)$ we use $\omega_p \approx 1.4$ eVfor both crystals.

V. EXTENDED DRUDE ANALYSIS: INTERBAND CONTRIBUTIONS AND RANGE OF VALIDITY.

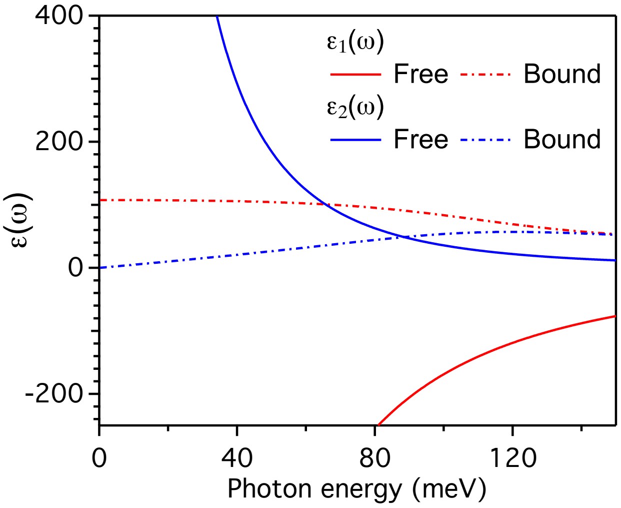

FIG. S5: (Color online) **Dielectric response.** Decomposition of the dielectric function in bound and free charge response at 40 K corresponding to the model parameters of table S1.

As discussed in the main text, overlapping intra- and interband conductivities complicate the extraction of the memory function from optical conductivity data. In the absence of overlapping intra- and interband conductivities

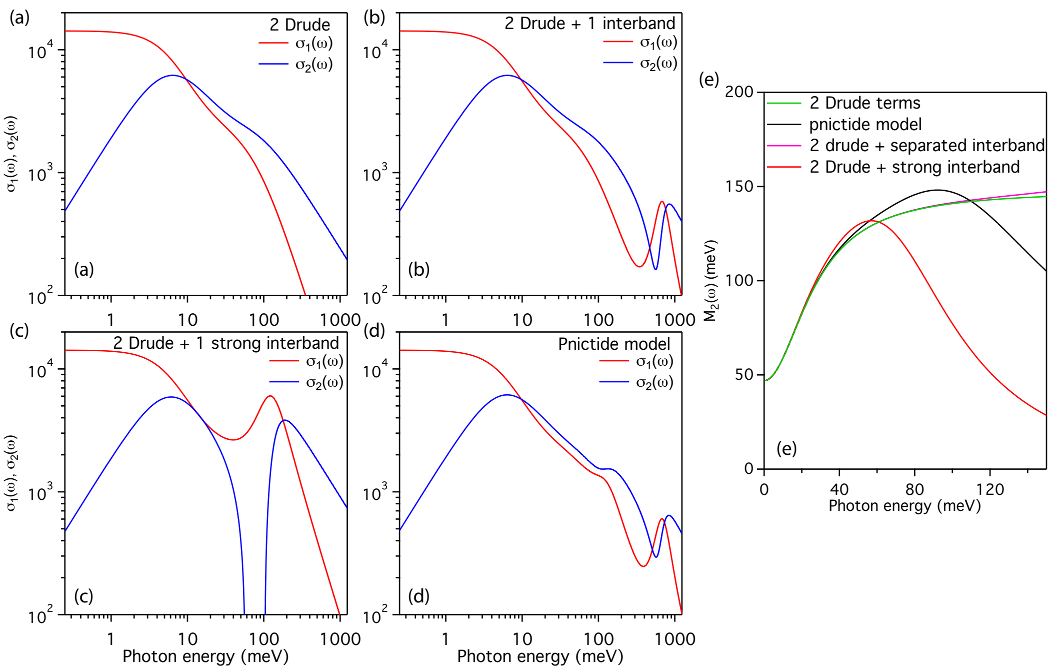

FIG. S6: (Color online) Model optical conductivity for (a): superposition of two Drude terms, (b): two Drude terms and a well separated interband transition, (c): two Drude terms and a strong interband transition overlapping with the intraband response and (d): a model used for the annealed crystal. (e): Memory functions calculated for each of the panels (a-d) without taking the presence of the interband transitions into account. The comparison shows that the additional frequency dependence of interband processes is modest for photon energies up to 50 meV for the worst case considered (e.g. two Drude terms + overlapping strong interband transition).

(e.g. such as is the case for cuprate HTSC) one can write the memory function in terms of the dielectric function:

$$M_1(\omega) = \frac{\omega_p^2}{\omega} \frac{\varepsilon_2(\omega)}{[\varepsilon_{\infty,IR} - \varepsilon_1(\omega)]^2 + \varepsilon_2^2(\omega)} \tag{S2}$$

$$M_2(\omega) = \frac{\omega_p^2}{\omega} \frac{\varepsilon_{\infty,IR} - \varepsilon_1(\omega)}{[\varepsilon_{\infty,IR} - \varepsilon_1(\omega)]^2 + \varepsilon_2^2(\omega)} - \omega \tag{S3}$$

where $\varepsilon_{\infty,IR}$ represents a frequency independent contribution to the *real part* of the dielectric function due to high energy interband transitions, which can be estimated from the oscillator strengths of those transitions. What happens in the case of iron-pnictides where interband transitions have a low energy onset (estimated to be situated around 100 meV, see fig. S3)? This is most clearly illustrated in figure S5 where we show the free charge (or intraband) and bound charge (or interband) contributions to the dielectric model presented in table S1. The question that arises is whether the bound charge response can be approximated with a constant in the photon energy range where we want to analyze the memory function. Given the relative strengths of the free and bound charge response, the approximation of using a frequency independent $\varepsilon_{\infty,IR}$ could possibly be upheld below about 50 meV. This is fortified by explicitly calculating the imaginary part of the memory function for a series of models as we will now discuss.

Fig. S6 shows the optical conductivity for 4 different models. In panel S6a the real and imaginary part of a sum of two Drude terms with different widths is shown. Panel S6b,c show the same model but now with a single interband transition added to it. In panel S6b the intra- and interband parts are well separated as in the cuprates, while panel S6c has a strong interband transition well within the intraband region. Finally, panel S6d shows the optical conductivity for a model similar to the pnictide model. Panel S6e now compares the extracted memory functions for these models *without* making any correction for the interband contribution. The 2 Drude case (in green) would represent the correct optical scattering rate that we would like to extract in an experiment. The other cases show deviations from this ideal curve to varying degrees. What is important for the current work is that below about 50 - 80 meV the frequency dependence in all cases is very close to the ideal case indicating that in the realistic case relevant to the iron-pnictides (black curve) the extended Drude model gives relevant results in the photon energy range discussed in the main text.

VI. EXTENDED DRUDE MODEL: COMPARISON OF METHODS

To test the robustness of the results presented in the main text we used two methods to determine the memory function. In the first method we approximated the interband contribution with a temperature independent $\varepsilon_{\infty,IR} \approx$ 100 - 105 (for as-grown and annealed crystals respectively). We then used Eq. S2 to calculate the memory function. In the second method we subtracted the interband part obtained from the Drude - Lorentz model obtained at each individual temperature. The results of the second method are presented in the main text and the results of the first method are presented in fig. S7. At low temperatures ($\leq$ 150 K) and photon energies between 10 - 50 meV both

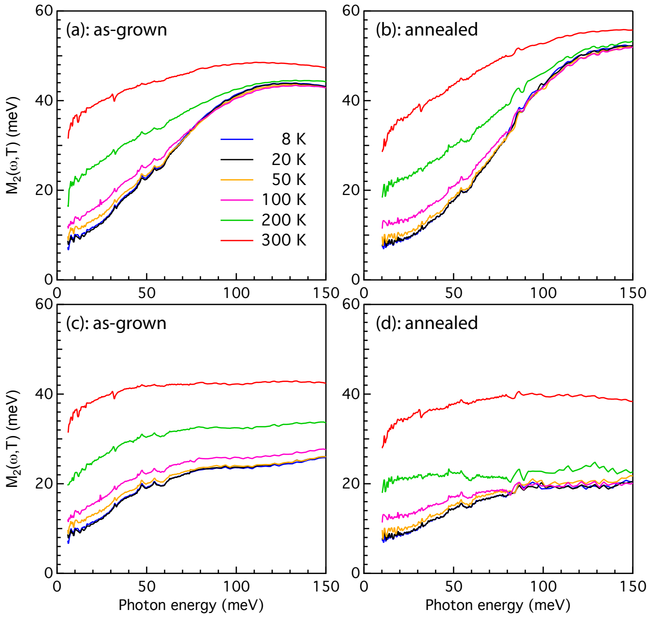

FIG. S7: (Color online) **Memory functions.** Comparison between the memory functions obtained using the method of subtracting $\varepsilon_{\infty,IR} \approx$ 100 -110 (a,b) and by subtracting the full frequency dependent interband conductivity (c,d).

methods give nearly identical results. At higher temperatures differences are starting to become more evident as can be seen most clearly by comparing the 200 K data for the annealed crystal. Note that the main result of our article, namely the T^2 and ω^2 dependence of $M_2(\omega, T)$, would be extended over a larger energy and temperature range if we use the first method to determine $M_2(\omega, T)$ but with a somewhat smaller value of p. Next we discuss the estimation of the value of p for which all the data collapses onto a universal curve. In ref. [4] the following method was proposed: one plots the data as function of $\xi^2 = \left[(\hbar\omega)^2 + (p\pi k_B T)^2\right]$ for a range of values of p. We take $1 \leq p \leq 2$ with steps of 0.01. One then calculates the root-mean square for each value of p determined by summing over the deviations of each temperature from a universal curve for that value of p. These RMS values are then summed over a range of temperatures up to a certain maximal temperature. Figure S8 shows the dependence of p on the maximum temperature, T_{max}, used in the scaling analysis. We apply this method to the memory function extracted with both methods indicated above and find that the value of p depends weakly on temperature. At 100 K, where the power of the frequency dependence starts to deviate from $\eta \approx 2$ we find the values $p = 1.2$ (ε_∞ correction) and $p = 1.47$ (interband subtraction).

In the main article we showed that the optical data can be scaled as a function of ω^2 and T^2. In particular, we showed that the DC extrapolation of the scattering rate follows a T^2 temperature dependence, implying that the resistivity is also a function of T^2. To confirm this expectation we show in figure S9a the resistivity data of fig. S1 plotted as function of T^2. We indeed find that $\rho(T)$ is an approximate function of T^2 in the same range of temperatures (30$\leq$ T $\leq$120 K) as $1/\tau(0)$ (see fig. S9b). From fig. S9a we estimate $d\rho/d(T^2) \approx 6.5{\cdot}10^{-9}\ \Omega cm/K^2$, while $d(\tau)^{-1}/d(T^2) \approx 3.5{\cdot}10^{-3}\ cm^{-1}/K^2$. We can now use the Drude expression for the DC resistivity $\rho = 4\pi/\omega_p^2\tau$ to compare the slopes of both quantities. Together with the plasma frequency $\omega_p \approx 11290\ cm^{-1}$ we obtain $d\rho/d(T^2) \approx 1.7{\cdot}10^{-9}\ \Omega cm/K^2$ from $d(\tau)^{-1}/d(T^2)$. Given the uncertainties involved in determining $1/\tau(0)$ this is a reasonable agreement. We note that the resistivity deviates from the approximate T^2 behaviour at the onset of superconductivity and at elevated temperatures. Above the temperature scale where Fermi liquid scaling applies both the DC resistivity and scattering rate are still approximate functions of T^2, but with slightly smaller slopes as can be seen from the deviation from the black lines.

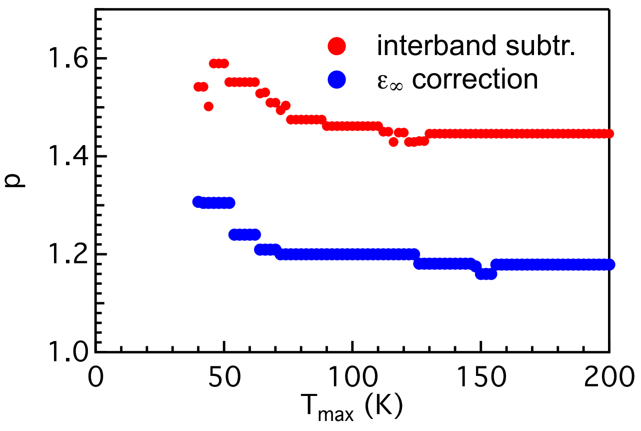

FIG. S8: (Color online) **Minimum p values.** Value of p giving the best scaling collapse as a function of maximum temperature used in the determination of the deviation of the data from a universal curve.

Figure S9(c) we show the temperature dependence of the prefactor, B(T), of the frequency component appearing in $M_2(\omega, T) = 1/\tau(0, T) + B(T)\omega^{\eta(T)}$. Comparing this empirical relation with Eq. 2 of the main manuscript, B(T) is expected to be temperature independent in the range of validity of Eq. 2. Fig. S9(c) shows that this is indeed the case below $T \approx 100$ K. In panel S9(d) we plot the scaling collapse for the memory function obtained by subtracting $\varepsilon_{\infty,IR}$. Note that in this case the scaling extends over a larger energy window due to the higher energy where the memory function saturates (≈ 120 meV, fig. S7b).

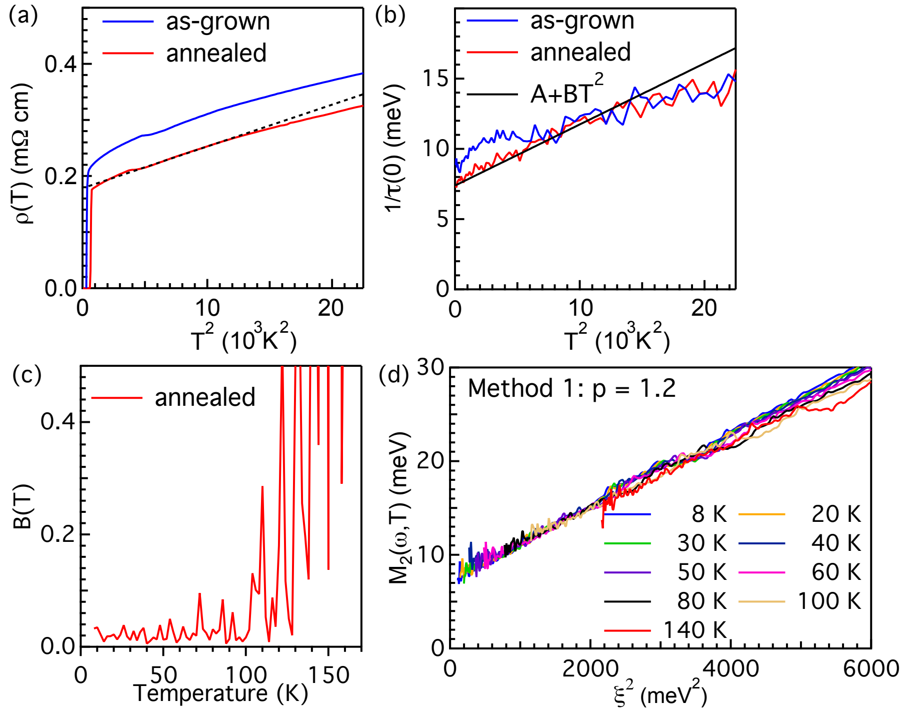

FIG. S9: (Color online) **Scaling analysis.** (a) Resistivity as function of T^2. The dashed line is a guide to the eye. (b): DC scattering rate as function of T^2. This panel is equivalent to Fig. 1e of the main text (c): Prefactor, B(T), of the frequency component of the memory function. (d): Scaling collapse of $M_2(\omega, T)$ obtained by correcting M_2 with a frequency independent interband contribution.

VII. ZERO CROSSINGS OF $\Delta\sigma(\omega)$ IN THE PRESENCE OF INTERBAND TRANSITIONS

In reference [5] it was shown that in a Fermi liquid the optical conductivity could be described by three different frequency regimes. These regimes are, at low temperature, separated by crossings of $\sigma_1(\omega)$ and $\sigma_2(\omega)$. It was shown that at low frequency the optical conductivity follows a Drude behaviour, with $\sigma_1(\omega) \leq \sigma_2(\omega)$, at intermediate frequency a thermal regime appears with $\sigma_1(\omega) \geq \sigma_2(\omega)$ and finally at high frequency $\sigma_1(\omega) \leq \sigma_2(\omega)$ again. The boundary separating these regimes can be easily derived from the optical conductivity. We have:

$$\sigma(\omega) = \frac{i\omega_p^2}{4\pi}\frac{1}{M(\omega)+\omega} \tag{S4}$$

with $M(\omega)$ given by [5],

$$M(\omega) = \frac{i}{\pi k_B T_0}\left[\omega^2 + (2\pi k_B T)^2\right]. \tag{S5}$$

By equating $\sigma_1(\omega) = \sigma_2(\omega)$ one obtains a second order equation relating temperature T and frequency ω. Solving for T gives[5]:

$$T_1(\omega) = \sqrt{\frac{3k_B T_0}{8\pi}\left(\omega - \frac{2\omega^2}{3\pi k_B T_0}\right)}. \tag{S6}$$

As was shown in ref. [6], the pre-factor of the temperature term can be different from 2 if additional contributions to the frequency dependence of the self-energy are present, in which case:

$$\Sigma(\omega) = \frac{i}{\pi k_B T_0}\left[(1+a)\omega^2 + (\pi k_B T)^2\right]. \tag{S7}$$

Given the value of $p = 1.47$ obtained in our study, we should therefore take,

$$T_1(\omega) = \sqrt{\frac{3k_B T_0}{8\pi}\left(\omega - \frac{2(1+a)\omega^2}{3\pi k_B T_0}\right)}. \tag{S8}$$

with $a = (p^2-4)/(1-p^2)$. In the iron-pnictide superconductors a low energy interband contribution to the optical conductivity further complicates matters. In the article we therefore use the full Allen-Kubo formula and calculate $\Delta\sigma(\omega, T)$, including the full frequency dependent interband conductivity. It is however instructive, and useful for other materials, to approximate the interband contribution with a purely reactive component and derive an analytic expression for the zero crossings. As shown in the main text the zero crossings of this expression and the full calculation do not differ too much. We therefore start from,

$$\sigma(\omega) = \frac{i\omega_p^2}{4\pi}\frac{1}{M(\omega)+\omega} - \frac{i\omega\varepsilon_\infty}{4\pi} \tag{S9}$$

which is equivalent to,

$$\sigma(\omega) = \frac{1}{4\pi}\left[\frac{\omega_p^2 M_2(\omega) + i\left[\omega_p^2\bar{\omega} - \omega\varepsilon_\infty\left(\bar{\omega}^2 + (M_2(\omega))^2\right)\right]}{\bar{\omega}^2 + (M_2(\omega))^2}\right] \tag{S10}$$

with $\bar{\omega} = \omega + M_1(\omega)$. We consider again the case where $M_1(\omega)$=0 and $M_2(\omega) \propto \left[(1+a)\omega^2 + (2\pi k_B T)^2\right] + \Gamma_0$. Here the last term is an additional, frequency independent impurity scattering rate. Solving Eq. S10 for $\sigma_1(\omega) = \sigma_2(\omega)$ results in a fourth-order equation in T and ω, which has only one physical solution:

$$T_\infty(\omega) = \frac{1}{4}\sqrt{\frac{3}{\varepsilon_\infty \pi\omega}\sqrt{T_0^2\left[\omega_p^4 + 4\varepsilon_\infty\omega^2\omega_p^2 - 4\varepsilon_\infty^2\omega^4\right]} - \frac{4\omega^2(1+a)}{\pi^2} - \frac{3T_0\omega_p^2}{\varepsilon_\infty\pi\omega} - \frac{6\Gamma_0 T_0}{\pi}}. \tag{S11}$$

Although this expression looks more unwieldy than Eq. S6, most of the parameters can be determined independently from each other. The plasma frequency, ω_p, can be determined from a spectral weight analysis, while ε_∞ can be estimated from a Drude-Lorentz analysis. Γ_0 can be determined at low temperature from an extrapolation of $M_2(\omega)$ to $\omega = 0$. This leaves T_0 and p as free parameters.

VIII. SELF-ENERGY OF A LOCAL FERMI LIQUID WITH A CUTOFF.

The imaginary part of the complex self-energy $\Sigma(\omega,T) = \Sigma_1(\omega,T) + i\Sigma_2(\omega,T)$ of a local Fermi liquid is given by [5, 6],

$$\Sigma_2(\omega,T) = \begin{cases} \frac{1}{\pi k_B T_0}[(1+a)(\hbar\omega)^2 + (\pi k_B T)^2] & 0 < |\omega| < \omega_c \\ \frac{1}{\pi k_B T_0}[(1+a)(\hbar\omega_c)^2 + (\pi k_B T)^2] & \omega_c < |\omega| < D \\ 0 & D < |\omega| \end{cases}, \tag{S12}$$

where D represents (a fraction of) the total bandwidth. An analytical expression for the real part of the self-energy can be obtained by Kramers-Kronig transformation. It is given by

$$\Sigma_1(\omega,T) = \frac{(1+a)}{\pi^2 k_B T_0} 2\omega\omega_c + \frac{(1+a)}{\pi^2 k_B T_0}(\omega_c^2 - \omega^2)\left[\log\left(\frac{|D-\omega|}{|\omega_c-\omega|}\right) + \log\left(\frac{|\omega_c+\omega|}{|D+\omega|}\right)\right] + \frac{\Sigma_2(\omega,T)}{\pi}\log\left(\frac{|D-\omega|}{|D+\omega|}\right) \tag{S13}$$

for $0 < |\omega| < \omega_c$,

$$\Sigma_1(\omega,T) = \frac{(1+a)}{\pi^2 k_B T_0} 2\omega\omega_c + \frac{(1+a)}{\pi^2 k_B T_0}(\omega^2 - \omega_c^2)\log\left(\frac{|\omega_c-\omega|}{|\omega_c+\omega|}\right) + \frac{\Sigma_2(\omega_c,T)}{\pi}\log\left(\frac{|D-\omega|}{|D+\omega|}\right) \tag{S14}$$

for $\omega_c < |\omega| < D$, and

$$\Sigma_1(\omega,T) = \frac{(1+a)}{\pi^2 k_B T_0} 2\omega\omega_c + \frac{\Sigma_2(\omega,T)}{\pi}\log\left(\frac{|\omega_c-\omega|}{|\omega_c+\omega|}\right) + \frac{\Sigma_2(\omega_c,T)}{\pi}\left[\log\left(\frac{|D-\omega|}{|\omega_c-\omega|}\right) + \log\left(\frac{|\omega_c+\omega|}{|D+\omega|}\right)\right] \tag{S15}$$

for $\omega > D$. The real and imaginary parts of this self-energy are plotted in Fig. 3a of the main text.

---

[1] *http://optics.unige.ch/alexey/reffit.html.*
[2] A. B. Kuzmenko, Review of Scientific Instruments **76**, 083108 (2005).
[3] D. Wu, N. Barišić, P. Kallina, A. Faridian, B. Gorshunov, N. Drichko, L. J. Li, X. Lin, G. H. Cao, Z.-A. Xu, et al., Physical review. B, Condensed matter **81**, 100512 (2010).
[4] D. Stricker, J. Mravlje, C. Berthod, R. Fittipaldi, A. Vecchione, A. Georges, and D. Van Der Marel, Physical Review Letters (ISSN 0031-9007) **113**, 087404 (2014).
[5] C. Berthod, J. Mravlje, X. Deng, R. Zitko, D. Van Der Marel, and A. Georges, Physical review. B, Condensed matter **87**, 115109 (2013).
[6] D. L. Maslov and A. V. Chubukov, Physical review. B, Condensed matter **86**, 155137 (2012).
